# Bridging Neuroscience and Machine Learning: A Gender-Based Electroencephalogram Framework for Guilt Emotion Identification

**DOI:** 10.3390/s25041222

**Published:** 2025-02-17

**Authors:** Saima Raza Zaidi, Najeed Ahmed Khan, Muhammad Abul Hasan

**Affiliations:** 1 CS & IT Department, NED University of Engg & Tech, Karachi 75270, Pakistan; najeed@cloud.neduet.edu.pk; 2 Bio-Medical Enginering Department, NED University of Engg & Tech, Karachi 75270, Pakistan; abulhasan@cloud.neduet.edu.pk

**Keywords:** emotion recognition, brain–computer interaction, machine learning, guilt, EEG

## Abstract

This study explores the link between the emotion “guilt” and human EEG data, and investigates the influence of gender differences on the expression of guilt and neutral emotions in response to visual stimuli. Additionally, the stimuli used in the study were developed to ignite guilt and neutral emotions. Two emotions, “guilt” and “neutral”, were recorded from 16 participants after these emotions were induced using storyboards as pictorial stimuli. These storyboards were developed based on various guilt-provoking events shared by another group of participants. In the pre-processing step, collected data were de-noised using bandpass filters and ICA, then segmented into smaller sections for further analysis. Two approaches were used to feed these data to the SVM classifier. First, the novel approach employed involved feeding the data to SVM classifier without computing any features. This method provided an average accuracy of 83%. In the second approach, data were divided into Alpha, Beta, Gamma, Theta and Delta frequency bands using Discrete Wavelet Decomposition. Afterward, the computed features, including entropy, Hjorth parameters and Band Power, were fed to SVM classifiers. This approach achieved an average accuracy of 63%. The findings of both classification methodologies indicate that females are more expressive in response to depicted stimuli and that their brain cells exhibit higher feature values. Moreover, females displayed higher accuracy than males in all bands except the Delta band.

## 1. Introduction

Acceptance and amelioration of emotion-aware computing requires computers to be capable of interpreting and understanding a variety of human emotions. This understanding enables computers to respond and act more effectively, thereby maximizing user satisfaction. Emotions have a major role due to their significant effect on human moods, decisions and regular communication. Therefore, it is critical for computers to efficiently recognize human emotions to facilitate better integration into daily life. This capability helps machines and computers to be more attentive to human emotional needs. Due to the rapid growth of affective computing, emotion-aware systems are extensively used in real-time systems in health facilities [1,2,3,4], smart driving systems [5], voice recognition systems [6], humanoids [7,8], intelligent tutoring systems and education [9,10,11]. Therefore, extensive research is required in the field of affective computing to design and build user-friendly, effective, intelligent and reliable emotion identification systems. These emotion identification systems should ensure accuracy, robustness and adaptability to real-time applications. More than 90 definitions of human emotion [12] have been reported in the literature, and emotions are classified into primary and secondary emotions [13,14,15,16,17,18,19]. Significant studies of emotion recognition have focused on primary emotions. However, few researchers have paid attention to secondary complex emotions such as guilt, terror, or confusion. These secondary emotions are understudied compared to the primary ones.

The analysis of human emotion identification systems has gained remarkable importance in recent years [20,21]. Researchers have explored various dimensions of human–machine interaction to address potential challenges in communication with hominids. Researchers have tried to enable computers to handle the affective needs of human beings by observing different modalities. These modalities include human facial expressions [9,22,23,24], handwriting style [25], associated physiological signals or brain activities [26,27,28], gesture recognition [29,30] and human speech [31,32,33]. Nonetheless, these factors may be unreal, posed, disguised or camouflaged, easily excluding physiological signals or brain activities. Lindquist et al. [34] suggest that the human brain plays a pivotal role in the human emotional paradigm. EEG signals are titled as a “window on the mind” [35] due to their genuine nature as they cannot be falsified. This supports the application of brain–computer interaction technology to enhance emotion-aware computing. In recent years, brain signal acquisition devices have attracted researchers, such as EEG signals, Galvanic Skin Response (GSR) and many others. EEG is one of the most prominent and reliable modalities to enhance affective computing studies [22,23,36].

**Figure 1:**
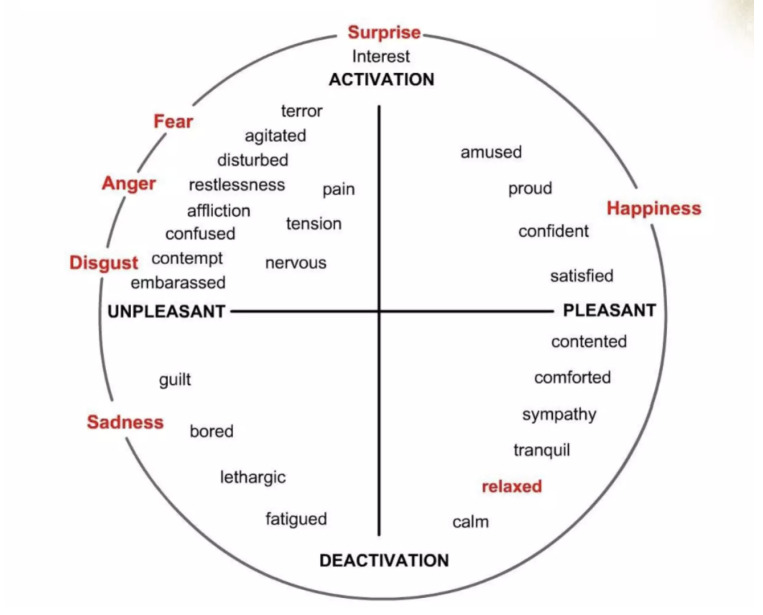
Russel Circumplex Emotion Model [18].

The proposed research investigates the EEG signals linked to humans’ guilt state and explores the impact of gender differences on the expression of guilt and neutral emotions in response to depicted stimuli. As per our knowledge, EEG data related to guilt and its gender differences have never been explored or analyzed before this contribution. The proposed methodology contributes to the fields of human emotions, behavior and human–machine interaction by highlighting insights of a secondary emotion [18] and the physiological signals linked to it. Guilt is a secondary emotion as shown in Figure 1, initiated by transgressions [37] and still needs attention from scholars despite its high involvement in psychiatric disorders.

Guilt plays an important role in self-improvement and strengthens multiple relationship-enhancing factors. It is also critical for improving interpersonal relationships. People experiencing guilt often isolate themselves from their community, which may result in society losing their significant contributions. If a machine automatically detects a person feeling guilty, it may aim to boost their morale and support self-improvement. Guilt is directly linked with feelings of depression and anxiety. Machines’ ability to identify guilt-related EEG patterns may aid an individual’s well-being. Guilt shapes the future behavior of a person; thus, a machine that detects guilt-related EEG signals may provide better responses to individuals.

This study provides a state-of-the-art contribution to emotion-aware computing specifically toward intelligent tutorial systems and clinical psychology. The remaining sections of the paper are presented as follows. Section 2 presents a description of existing emotion recognition systems. Section 3 demonstrates the proposed emotion identification system. Section 4 shows the performance and evaluation of the proposed methodology. Finally, in Section 5, the conclusion and future research directions are given.

## 2. Literature Review

Prior research advocates for using numerous machine learning algorithms for emotion identification from EEG signals. This section evaluates existing AI methods for recognizing emotions from EEG physiological signals. Gao et al. [38] employed the Phase Locking Value (PLV) method and developed the brain functional adjacency matrix of the EEG brain signal of each subject. For the decomposition of signals, the tucker decomposition technique was applied. Differential entropy and energy features were combined with dynamic brain functional connections and fed to the SVM classifier. The proposed system was validated on the ERN and DEAP data sets. Goshvarpour et al. [39] validated the proposed system on the DEAP data set. The proposed system fed cross-information potential (CIP) between two electrodes to SVM and kNN classifiers. kNN outperformed the SVM classifier with a maximum frequency of 90%, and the Gamma frequency band played a pivotal role in low–high valance and arousal classification. Liu et al. [40] demonstrated an EEG-based emotion identification technique on a DEAP data set using a pre-trained convolution capsule network developed on an attention mechanism. The EEG features were extracted with pre-trained Mobile Net from pre-processed EEG data. The proposed methodology was evaluated in two dimensions: subject-dependent and subject-independent. This algorithm identified the valence and arousal of the data set. Tuncer et al. [41] applied a fractal pattern approach and Q-factor Wavelet Transform (TQWT) for feature extraction and signal decomposition, respectively. The calculated features were tested on the GAEMO EEG data set using Linear Discriminant Analysis (LDA), K-Nearest Neighborhood (k-NN) and support vector machine (SVM). The results presented that SVM outperformed the LDA and KNN. Li et al. [42] developed EEG-based emotion recognition by first extracting meaningful information from EEG data by learning them simultaneously through MIL (multi-task learning) and then usin a capsule network combined with an attention mechanism to identify arousal, valence and dominance in the DEAP and DREAMER data sets. Joshi et al. [43] extracted Power Spectral Density, Hjorth parameters, differential entropy and linear formulation of differential entropy from DEAP, SEED and the collected data set. The extracted features were then fed to a deep RNN model based on a bidirectional long short-term memory (BiLSTM) network, Multilayer perceptron (MLP), K-Nearest Neighbors (k-NN) and support vector machine (SVM). As per the evaluation, optimized BiLSTM performed the best among all the classifiers. Gao et al. [44] calculated both time-domain features listed as Hjorth, differential and sample entropy, and frequency-related features as Power Spectral Density. These extracted features were fused with fully connected layers and the SVM classifier was applied to classify the EEG data. The proposed algorithm was evaluated on a publically available data set, DEAP, achieving accuracies of 80.52% for valence and 75.22% for arousal. Yuvaraj et al. [45] tested two classifiers, a support vector machine (SVM) and a Classification and Regression Tree (CART), on five publicly available data sets—MAHNOB-HCI, DEAP, SEED, AMIGOS and DREAMER. The result showed that the FD-CART feature classification method outperformed the SVM classifier. Subasi et al. [46] applied Tunable-Q Wavelet Transform (TQWT) to find the required features from a publically available data set (SEED). Data were de-noised by applying a Symlets-4 filter. In the classification phase, the rotation forest ensemble (RFE) classifier was utilized with multiple algorithms such as k-NN, SVM, ANN and many more. The result demonstrated that the combination of REF and SVM classifiers performed the best.

## 3. Proposed Methodology

The proposed methodology separates guilt and neutral emotion EEG data. The emotions can be induced among participants by using different emotion induction techniques. Due to the unavailability of stimuli for guilt and neutral emotion induction, guilt stimuli images were prepared in the form of storyboards. The design and development of these stimuli images were based on a literature review of Bhushan et al. [47] study, that developed comic sketches after collecting stories from participants.

### 3.1. Stimuli Set Construction for Target Emotion Induction

**Figure 2:**
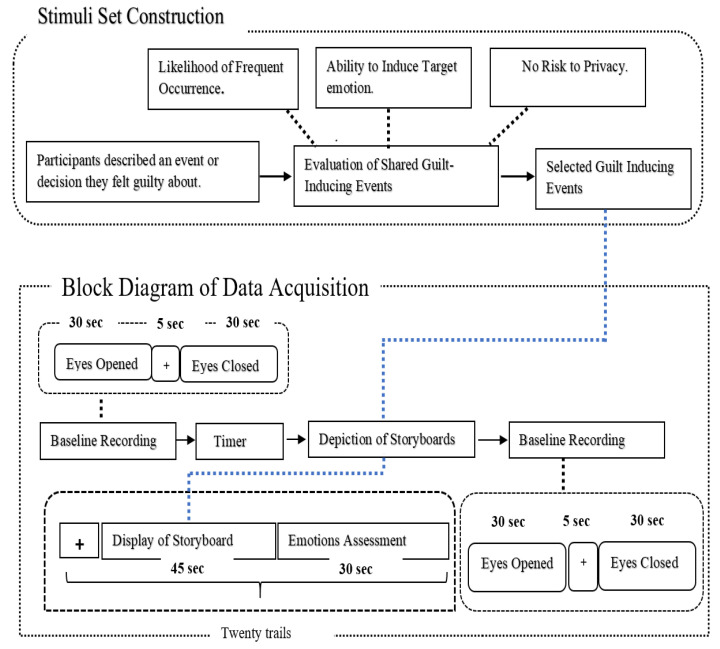
Block Diagram of Experimental Process: From Stimuli Development to Data Acquisition.

For Stimuli Set Construction, storylines were developed with the help of 52 volunteer participants. The age group of participants was 19–22 (mean = 20.5 years, SD = 1.6). They were Pakistanis from middle-class backgrounds. All the participants were healthy and university students. Participants were required to fill out a consent form that was reviewed and accepted by the Ethical Committee of NED University of Engineering and Technology. The block diagram illustrating the Stimuli Set Construction phase, along with the Data Acquisition phase, is presented Figure 2.

#### 3.1.1. Phase I: Development of Storyline

**Figure 3:**
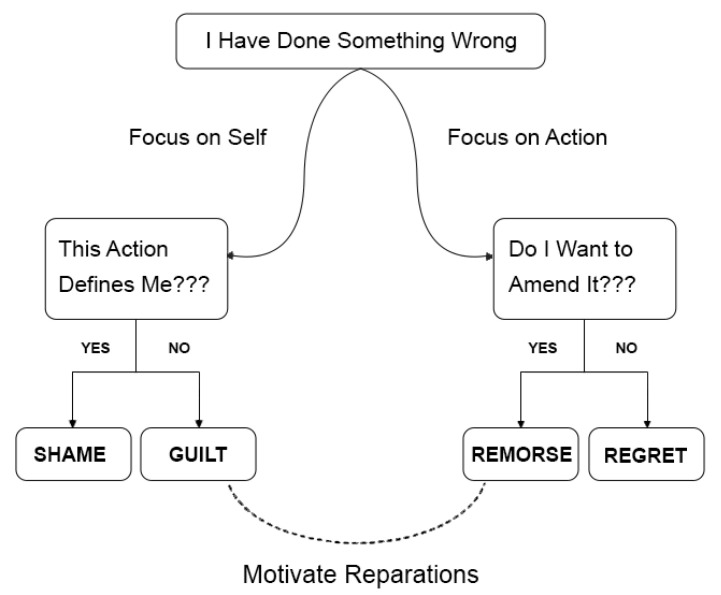
Illustration of Guilt, Shame, Regret and Remorse [48].

Before the storyline development phase, participants were guided about guilt and neutral emotions in detail. The difference between guilt and shame was also discussed in detail. Additionally, they were also guided about feelings of regret and remorse, as remorse is very much connected to guilt. This paper follows the definition of guilt as “An emotion which compels the wrongdoer to improve their actions in future and repair their crashed relations” [48,49]. This is in contrast to shame, where a person tries to withdraw from social contacts after wrong deeds [48,50,51,52]. With shame, a person considers themselves a bad person. The link between guilt, shame, remorse and regret is illustrated in Figure 3.

After answering all their queries, some sample stories taken from the studies [47,53,54] were shared with them. This was to give them a basic idea of the type of stories they could share with us. These instructions were explained to the participants together; however, the story-sharing process was conducted separately with a group of 15 participants at a time. During the story-writing phase, participants were seated at a distance from one another. The story collection process was completed in a single day.

Participants were asked to recall an event or decision that might ignite feelings of guilt in them. They were guided to write about this in as much detail as they felt comfortable. This phase provided a total set 52 guilt-related events shared by participants.

#### 3.1.2. Phase II: Selection of Events for Storyboard Development

The shared events were evaluated based on the probability of inducing the target emotion and the frequency of occurrence, so another person can relate to it easily; it was ensured that selected events did not reveal anything personal about the storyteller. These measurement criteria were related to the instructions given to the participants earlier before the writing events. At the end of this phase, almost 10 storylines were selected for further development (see Table 1).

#### 3.1.3. Phase III: Storyboard Construction for Guilt and Neutral Emotion Induction

**Figure 4:**
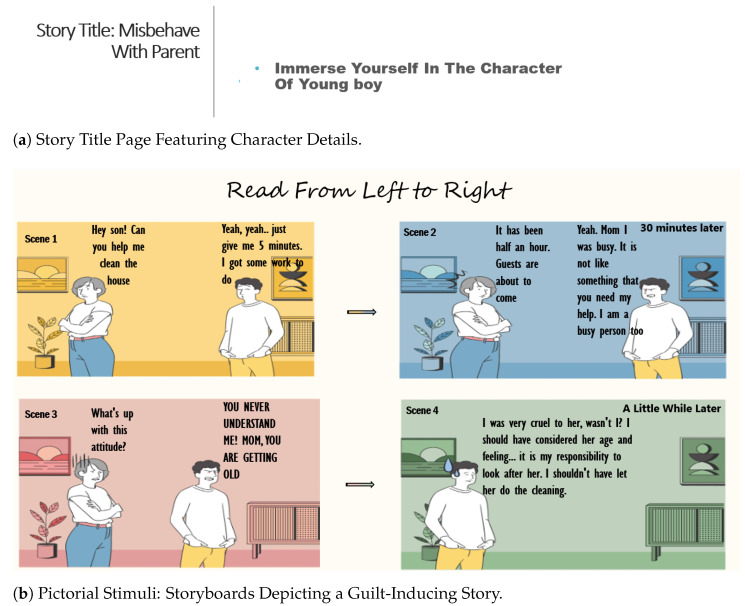
Title Page and Stimuli Storyboard.

After the selection of storylines, 10 story boards were developed based on the guilt-provoking events. These events were shared by the participants in the previous “Development of Storyline Phase”. These 10 storyboards were developed using CANVA software available online for graphics designing. Developed storyboards (See Figure 4) were shown to participants during the data collection phase to induce guilt and neutral emotions among participants.

As per prior research, neutral emotion means the absence of all emotion. This is when a person feels no emotion, neither happy nor sad. In accordance with past studies, guilt has been widely explored with neutral or control conditions as suggested in [26,48,55,56,57,58,59]. Therefore, 10 events were selected for storyboard development for neutral emotion induction. A few of these events were selected from prior research [55,56,60], with a little description added that was required to prepare a complete story. A few events were based on daily routine life experiences. These events did not produce any positive or negative affect on the perceiver, e.g., going for shopping or preparing supper. Afterward, 10 storyboards were developed based on neutral events as shown in Table 2 in a similar way as they had been created earlier for guilt emotion induction.

### 3.2. Data Acquisition

The experiment was carried out in a controlled environment with low light and a noise-free environment. The workflow of the proposed methodology from Stimuli Set building to data acquisition is shown in Figure 2.

#### 3.2.1. Subject Details

There were a total of 16 participants, 8 females and 8 males, in the 18 to 24 age group, who participated in the study. Alarcao et al. [36] recommends that the median for the number of participants is 15 after exploring almost one hundred papers of EEG emotion recognition systems. In light of this finding and to ensure equal representation of both genders, the number of participants was set to 16. All were native healthy Pakistanis from middle-class backgrounds. They were required to fill out the consent form, with the ratification of the Ethical Committee of NED University of Engineering and Tech. The data of one participant were collected at a time. Only two participants were engaged in the data collection process on a single day. For better data acquisition, participants were requested to wash their hair before the data collection phases.

#### 3.2.2. Experiment Instructions

Subjects were given detailed guidance about guilt and neutral emotions. A similar explanation was provided to another group of participants during the storyline development phase. They were briefly guided about EEG equipment. Some sample storyboards were shown to them to help them feel at ease. Afterward, they were instructed regarding EEG recording as follows:

- To remain mentally and physically relaxed.
- To avoid blinking their eyes.
- To try to keep their eyes open.

#### 3.2.3. Experimental Protocol

**Figure 5:**
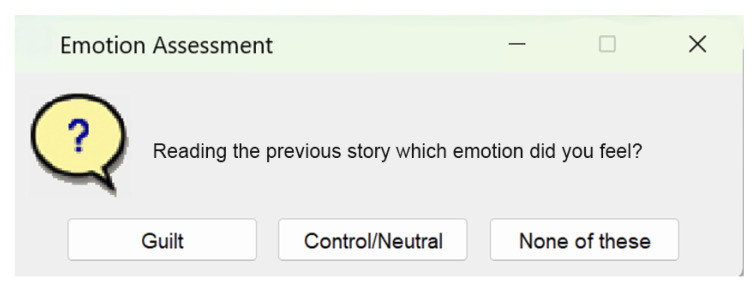
Emotion Assessment Dialogue Box Displayed After Each Stimulus.

The step-by-step experiment procedure is written below:

- First of all, a slide was exposed to the user for 5 s as a reminder message about performing fewer physical and eye movements.
- Afterwards, a baseline EEG was recorded for 65 s in which the first 30 s participants kept their eyes open. For the next 30 s, participants were prompted to keep their eyes closed. Between the eyes-open and eyes-closed sessions, a “+” sign was displayed for 5 s.
- On the next slide, a countdown timer from 5 to 1 s with a decrement of 1 s with each count was displayed. The timer was applied to boost the alertness level of the subject.
- Finally, the storyboards were displayed on the screen with a duration of 45 s for each storyboard. The first three seconds title of the story were shown with an instruction for viewers to immerse themselves in the mentioned character. After 3 s, a story with three to four scenes was depicted on screen for 42 s as shown in Figure 4.
- There were a total of 20 trials of storyboard depiction, with 10 storyboards for guilt emotion induction and 10 storyboards for neutral emotion induction. These storyboards were displayed in random orders using the randperm function of Matlab.
- After each trial, participants were asked to report the emotions they felt while looking at the depicted storyboards. As a result, collected data could be labeled as guilt, neutral and other, as illustrated in Figure 5. All emotion induction procedures include an emotion assessment feedback questionnaire to label emotions. This is due to the probability that the target emotion and similar situations may cause several emotions that vary from person to person [26,61]. SAM (Self-Assessment Manikin system) [62] is widely used in all emotion induction methodologies.
- In the end, after completing 20 trials, participants were again prompted for baseline recording for 30 s with their eyes closed and next 30 s with open eyes.

## 4. Data Processing

### 4.1. Data Sampling

In total, 16 participants were employed and each participant had twenty trials. In each trial, a stimulus storyboard was shown on screen for 45 s. There were 3 s for the title of the story and 42 s for the actual story board. Therefore, the total number of samples for each participant is as follows:

42 (story depiction time in sec) × 20 (num of trials) × 16 (num of channels)

The emotion assessment phase had three classes of emotion: guilt, control and none of the above. These categories were based on the participants’ selection of their felt emotion. After selecting the guilt and control classes only, total samples of both classes were:

146 samples of guilt

86 samples of control

where the dimension of each sample is 5209 by 16 as data are collected from 16 channel devices for 42 s with a frequency of 124 Hz,

where 42 × 124=5209

### 4.2. Data Pre-Processing and Segmentation

The recorded EEG data always have a high probability of being contaminated with unwanted data from numerous sources [63,64]. It is a challenging task to differentiate between artifacts and the original brain. Thus, proper data cleaning steps must be applied. At the first pre-processing stage, a minimum-order low-pass FIR filter was designed using the Kaiser Window method. The filter had a passband frequency of 0.15 Π rad/sample and a stopband frequency of 0.45 Π rad/sample. The passband ripple was set at 0.15, and the stopband attenuation was 65. Furthermore, the ICA technique was also applied to remove the eye artifacts, which are the primary noise source from recorded EEG data. ICA provides promising results when data sources are independent [65]. The major objective of ICA is to reduce the statistical dependence of the components on each other [66,67].

The filtered and ICA-pruned data set was then split into equal segments of a length of six seconds. Candra et al. [68] evaluated the emotion classification of EEG data using different time window lengths. The findings showed that time window lengths between 3 s to 12 s performed better than other window lengths. Before segmentation, the dimension of each sample was 5209 by 16; the last row of the data set was ignored to avoid complex calculation. A total of 86 control samples of dimensions 5208 by 16 were segmented. After dividing each sample into six segments of equal length, the total number of control samples was

86 × 6=512 samples of dimension 868 by 16.

where 512 is the number of samples, 868 is the number of rows and 16 is the number of channels. Similarly, in the case of the guilt class, after dividing each of the 146 samples into six segments of equal length 868 by 16, the total number of guilt samples was

146 × 6=876 samples of dimension 868 by 16.

### 4.3. Training Model

**Figure 6:**
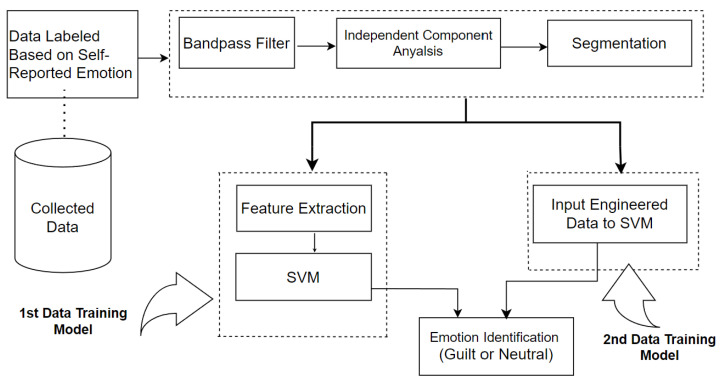
Block Diagram Illustration for Data Training.

To train the model, data were fed to train the SVM classifier in two different approaches, Data Engineering and Feature Computation, as shown in Figure 6.

#### 4.3.1. First Approach—Data Engineering

In the first approach, filtered, ICA-pruned and segmented data were loaded into the SVM classifier without extracting any features from it. This is a novel approach by using Data Engineering in an emotion classification system using machine learning. It treated chunks of data rows as feature sets themselves. This technique did not empower any single value feature to represent a large data set. In this model, all samples of EEG data linked to guilt emotions are called “Guilt samples (GSs)”. All samples of neutral or control emotions are called “Control Samples (CSs)”. The dimension of each sample is 868 by 16. Random samples from each emotion class were selected. These selected samples were then concatenated, forming data sets consisting of concatenated rows for each emotion class. After horizontally concatenating the selected samples, the rows for the guilt emotion class were combined and represented as “GAHC”. After horizontally concatenating the selected samples, the rows for the control emotion class were combined and represented as “CAHC”. The dimensions GAHC and CAHC depend upon the number of samples randomly selected as “R × 868 by 16”, where R represents the number of randomly selected samples. Overview of prepared data sets with their dimensions in Table 3.

**Classification to Recognize Emotions:** The developed GAHC and CAHC were then fed to the SVM classifier and trained. SVM is an extensively used supervised learning algorithm for emotion classification purposes [38,69,70]. SVM changes input data onto a higher dimensional feature space using the kernel transfer function; thus, the data become easy to separate in contrast to the original feature space. Here, the Radial Basis Function kernel function was utilized to map data onto a higher dimensional space. After training and learning the SVM classifier, a 5-fold technique was utilized. Prior research witnessed numerous values of “k” ranging from 2 to 10 in emotion classification systems. However, the proposed methodology chose k = 5, which has been applied in past research for emotion identification [71]. Moreover, Kusumaningrum et al. [72] demonstrated that 5-fold provided the best accuracy with multiple classifiers.

#### 4.3.2. Second Approach—Feature Computation

This traditional approach involved first applying wavelet decomposition to EEG signals. Features were then calculated from the detail coefficients and used as input for the classifier.

**Discrete Wavelet Transform:** At first, segmented data were decomposed into different frequency bands using Discrete Wavelet Transform (DWT). DWT serves as a reliable tool to analyze time-frequency domain information. DWT outperforms continuous wavelet transform in cleaning the redundant data [73]. DWT was utilized in several prior EEG emotion recognition systems [74,75]. The mathematical representation of DWT is expressed as:

(1) $$\gamma \left(y\right)=\int_{-\infty }^{\infty }x\left(y\right)\frac{1}{\sqrt{2^{a}}}\Psi \left(\frac{y-b\times 2^{a}}{2^{a}}\right)\mathrm{dy}$$

where Ψ is the mother wavelet, *a* and *b* are the scaling and shifting factors, respectively, and γ(y) is the Discrete Wavelet Transform (DWT) of x(y).

In this study, db3 was employed as the mother wavelet. The decomposition level was set to 6 to calculate detail coefficients at each level and the approximate coefficient with a sampling frequency is 124 Hz.

**Feature Extraction:** The feature extraction process represents raw data in numeral forms while preserving the meaning full information of actual data. This numerical information is further used to classify the data. As this is the first ever reported study on EEG signals and guilt emotion, the proposed methodology explores a wide range of EEG features to identify links between guilt and EEG signals. For the proposed methodology, selected features are written below.

**1. Entropy:**

Entropy is a measure of energy distribution for each band as shown below:

(2) $$\mathrm{Entropy}=-\sum_{i=1}^{n}\mathrm{pilog}10\mathrm{pi}$$

where pi is the probability of state i.

**2. Band Power:**

The Band Power value of signals was employed to classify the data, consistent with its application in prior research [76]. The Band Power (BP) is calculated as the addition of squares of total time domain samples divided by the total signal length.

**3. Hjorth Activity:**

Hjorth parameters were extracted from the segmented and pruned data set, following their common extraction in previous EEG-based emotion recognition systems [1,77]. Hjorth Activity is the squared standard deviation of the signal amplitude, represents variations in the signal power and is calculated as shown below:

(3) $$\mathrm{HjorthActivity}\left(a\right)=\frac{-\sum_{n=1}^{N}{\left(a\left(n\right)-\bar{a}\right)}^{2}}{N}$$

**4. Hjorth Complexity:**

This feature represents deviation in the signal frequency as shown below:

(4) $$\mathrm{HjorthMobility}\left(a\right)=\frac{\mathrm{Mobility}(a^{\prime })}{\mathrm{Mobility}(a)}$$

**5. Hjorth Mobility:**

This finds out the deviation in the power spectrum, as given below:

(5) $$\mathrm{HjorthMobility}\left(a\right)=\sqrt{\frac{\mathrm{var}(a^{\prime })}{\mathrm{var}(a)}}$$

Here, a’ is a derivate of signal a.

**Classification to Recognize Emotions:** All features were calculated separately for each frequency band, which were Alpha, Beta, Theta, Gamma and Delta. The calculated features were fed into the SVM classifier. The SVM classifier utilized the RBF kernel. The RBF kernel is much more efficient in separating non-linear relationships present in the data. The data set was input to the classifier using the k-fold technique. The k-fold technique splits data into a subset of approximately equal size. In each fold, one subset is used as the testing data set and the remaining four subsets serve as training sets. This classification process was repeated five times, taking each subset as the testing set only once.

### 4.4. Classification Using Deep Learning Approach

**Data Pre-Processing and Segmentation:** In the first step, a bandpass filter was applied on the data to eliminate noise. Low- and high-frequency artifacts were removed using a band bass filter of range 0.5 Hz and 50 Hz. Further data were normalized to zero mean and unit variance as the samples size was very small to be fed to deep learning. To make the data set sufficient for deep learning, EEG signals were segmented into smaller windows using a sliding window technique with a window length of 2 s, providing 248 samples per window given the 124 Hz sampling rate. The windows overlapped by 50%, in which each consecutive window shared 1 s of data with the previous window. This overlap provides more training samples from the original data. This enhances the model’s ability to generalize by providing it large numbers of samples.

**Model Architecture:** A 2D Convolutional Neural Network (CNN) was utilized for the classification. CNNs are efficient in learning both spatial and temporal patterns of EEG signals. The data were first converted into a 2D data model’s input shape. The input shape was defined as n channels, window size, 1, where n channels is the number of EEG channels, window size represents the number of samples per window and the depth is 1 as it is a single time series per window. To prevent overfitting, L1_L2 regularization was applied to the model’s weights. Moreover, to randomly eliminate half of the neurons, a 50% dropout rate was applied to further minimize the probability of overfitting. The model’s output layer utilized a softmax activation function that converted the network’s predictions into probabilities, enabling it to perform a binary classification task with two output classes (guilt and neutral).

**lModel Compilation:** Adam optimizer was utilized to compile the model with a loss function of sparse categorical cross entropy. The efficiency of the model is evaluated through the acquired accuracy to analyze the performance of model.

## 5. Result and Discussion

### 5.1. Classifier Performance—Data Engineering Approach

This methodology employed an engineered data set as a feature set and fed it to the SVM classifier. Several trials with different samples were performed to validate the model. For each trial, different sets of samples were randomly selected and concatenated. The findings of the initial trial are written below:

**Initial trial:** At first, a total of 16 random samples were selected from GAHC and CAHC, using the Matlab rand function. The number of samples selected was 16 because of the limited processing capacity of the hardware used for data processing. The selected samples for both CAHC and GAHC are shown in Table 4.

The selected samples were referred to as the data set after concatenation. These data sets were then fed into the SVM classifier. It provided promising accuracy in separating data of two separate classes. SVM employed a 5-fold method for this classification problem.

**Figure 7:**
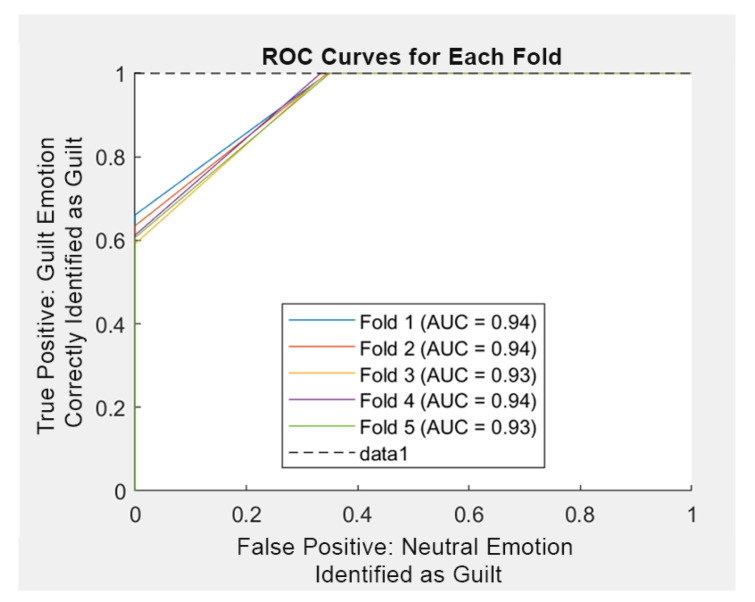
ROC Curve of Initial Trial.

Table 5 and the ROC curve in Figure 7, highlight performance assessments of the first trial. In the ROC curve, false positive (x-axis) defines the proportion of neutral emotions incorrectly identified as guilt. True Positive describes the proportion of guilt emotions correctly identified as guilt.

### 5.2. Classifier Performance of Multiple Trials—Data Engineering Approach:

**Figure 8:**
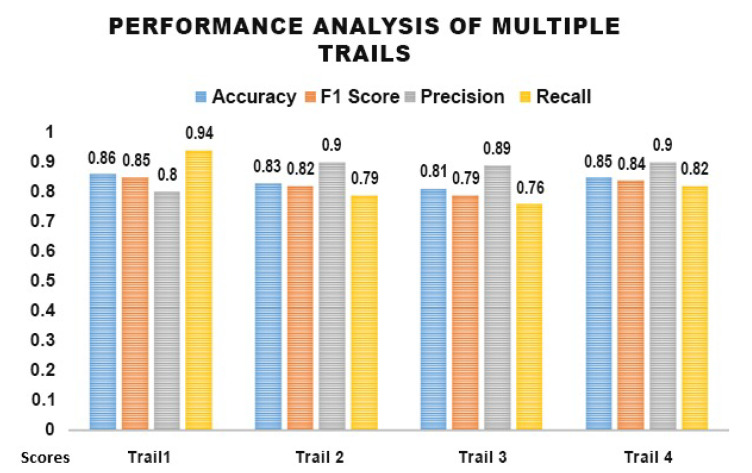
Performance Analysis of Multiple trials via Data Engineering Approach.

Multiple classification trials using 16 randomly selected samples were performed using the classifier. It provided an average accuracy of 83% across four trials. The performance of four trials is described in Figure 8.

The accuracy value indicates correctly classified instances and F1 Score describes a balance between precision and recall. Precision defines the values identified as positive and which are actually positive. Additionally, recall indicates the proportion of actual positive values identified correctly by the classifier. This metric presents a comprehensive view of the algorithm’s efficiency in multiple aspects.

The classifier’s results remained consistent across all four trials when employed using the provided data set. The algorithm efficiently and reliably classified data into two distinct emotional classes: guilt and neutral. These stable results indicate the classifier’s robustness and ability to maintain accuracy despite variations across multiple experimental trials.

### 5.3. Classifier Performance of Gender-Specific Data Engineering Approach

**Figure 9:**
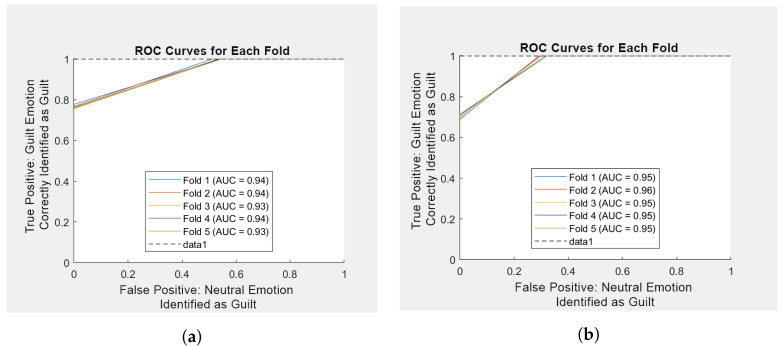
ROC curves. (**a**) ROC curve of Male Participants Data using Data Engineering Approach. (**b**) ROC Curve of Female Participants Data using Data Engineering Approach.

The proposed methodology evaluated the effect of stimuli and compared the differences in EEG data signals of male and female participants separately. Numerous studies have explored gender-related differences in emotion identification systems [78,79,80]. A total of 16 participants were employed in this study, 8 males and 8 females. A similar data processing and data-engineering approach was applied to both groups separately. The classification results provided an average accuracy of 88% for female participants and 80% for male participants, indicating a difference in classifier performance between genders. The cause of this difference may be subject to the more sensitive nature of the females surveyed. The Receiver Operating Characteristic (ROC) curves in Figure 9 presents the classification findings for both groups, highlighting a visual representation of the classifier’s discriminative ability across both groups. In the ROC curve, false positive (x-axis) defines the proportion of neutral emotions incorrectly identified as guilt. True Positive describes the proportion of guilt emotions correctly identified as guilt. Aligning with prior research, females display greater expressiveness in response to emotional stimuli.

### 5.4. Classifier Performance—Feature Extraction Approach

The second processing approach, feature extraction, provided a reasonable classification accuracy. The results show the model’s accuracy under two different feature fusion strategies: first, when features were calculated separately for each frequency band, and second, when extracted features from all frequency bands were combined into a single feature set. This traditional approach was evaluated using four performance metrics and is shown in Table 6. It achieved an average accuracy of 63%; precision was measured at 65%. The model’s recall reached 83% and F1 was calculated as 72%. This score indicates that the model maintained a good balance, favoring high recall while managing reasonable precision. The summary of classification accuracy achieved for each feature type (Hjorth parameters, Band Power, entropy and their combination) across different EEG frequency bands (Gamma, Beta, Alpha, Theta, Delta and All) is presented in Table 7. Hjorth Complexity, Mobility and entropy present a consistent accuracy of 62.9%, while Hjorth Activity and Band Power display more variability. Combining all features provides slightly better and more stable accuracy, demonstrating their complementary strengths.

### 5.5. Classifier Performance of Male and Female Participants—Feature Extraction Approach

When a similar approach was applied to the data of male participants and female participants, the findings demonstrated a notable variation in classification accuracy between the two groups. The method achieved an average accuracy of 60% for male participants and 68% for female participants. This difference was observed; however, the experimental procedure was identical for all participants. These findings are similar to prior studies [81,82] that females are more expressive and sensitive than males.

When the accuracies of both genders were compared for each band separately, it was concluded that female participants showed higher accuracies across all bands, except the Delta bandas shown in Table 8. This displays a distinct brain activity between males and females, specifically in lower frequency bands. Females’ brains might behave differently in ways that impacts Delta band activity, particularly in the case of complex emotions like guilt.

This suggests that female participants were more engaged and involved in the experiment than male participants, thus providing better accuracy.

### 5.6. Insights into Accuracy Across Data Engineering and Feature Extraction Approaches

**Figure 10:**
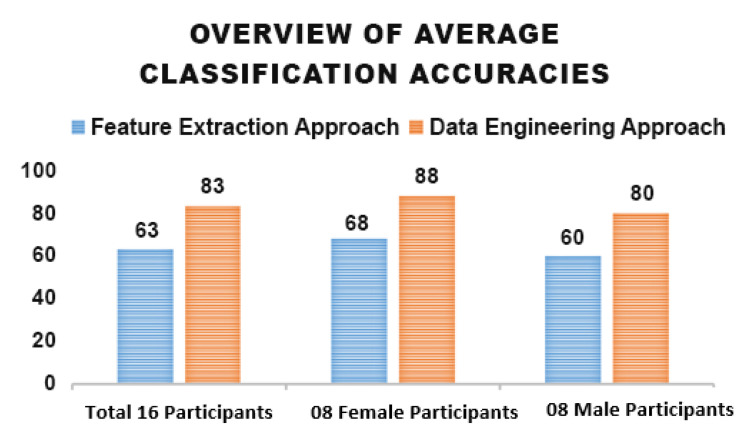
Average Accuracies Acquired by Data Engineering and Feature Extraction Approaches.

A comprehensive overview of accuracies acquired using both data training approaches is presented in Figure 10. These two approaches include the data engineering approach and feature extraction approach. The accuracies are highlighted on different subsets of data categorized as male and female participants. The respective strengths and weaknesses of data training in handling specific subsets of the data are highlighted in this figure given below. This comparison provides insights into the effectiveness of both approaches under different conditions and participant characteristics.

The findings of the emotion classification system demonstrate that the classifier provides better accuracy with an engineered data set, bypassing the feature extraction process from EEG data. The probable reasons behind the accuracy difference are as follows:

- A single value or a combination of a few values of calculated features represents a whole large data set of at least a 2 by 2 matrix or may be of more dimensions.
- These features may fail to preserve valuable information present in collected data and be unable to provide improved accuracy and results. As Rani et al. [83] discussed, feature extraction is a core challenge in affective computing.
- Due to phenomena of personal stereotypes where a system may rely on generalized assumptions about individuals [84], it is hard to extract features that perform efficiently over a large number of subjects. There is a probability that extracted features are unable to represent critical data points and information hidden in the EEG data set.
- Human physiological signals are complex with unidentified ranges, making it difficult to predict or standardize the data.
- The high probability of variation across several parameters further complicates the analysis, making it more challenging that few features can cover that variability.
- Decomposed frequency bands represent specific brain activities, and focusing only on these bands may lose critical information present outside these frequencies.

The aforementioned reasons could contribute to the significant improvement of 20% in classification accuracy that was achieved using the first approach—Data Engineering—where engineered data chunks were provided to the classifier.

### 5.7. Classifier Performance via CNN

To ensure the model’s robustness and prevent overfitting, k-fold cross-validation was utilized. K-fold splits the data set into five folds. In each fold, 80% of the data was used for training, and 20% was used for validation. This approach was repeated for all five folds, providing a reliable measure of model performance across different subsets of the data. The average accuracy achieved was 63%. There was no significant difference in accuracy achieved from deep learning.

### 5.8. Comparison with Existing Studies

It was challenging to compare the proposed algorithm with existing emotion recognition systems. Only two researchers have explored guilt emotion identification systems, each using different modalities. Differences in the data sets, stimuli used, recording devices, extracted features and classification methods made direct comparisons more challenging.

However, an overview of the existing studies investigating guilt, with details of the findings for each method, is demonstrated in Table 9. Key aspects of each study, such as data modalities and findings, are summarized in this table to highlight how the proposed methodology aligns with or varies from prior research.

Another approach to compare the proposed research with previous studies is to explore contributions within similar modalities (EEG signals), but investigating different emotions. By analyzing the similarities and variances in techniques and findings, meaningful insights can be drawn. Table 10 provides a comparative analysis of the proposed model with previous studies, that collected their own datasets. This ensures that the objective of proposed methodology is appropriately aligned with theirs. As a result, a more robust and direct comparison can be made. The studies shown in Table 10 utilized numerous classifiers for emotion identification; here, only those classifiers are mentioned that provided the best accuracy results among all. It is evident that SVM outperforms all classifiers.

As the proposed methodology also aimed to investigate the gender difference in response to stimuli, the findings of the proposed method can be compared with previous research on gender differences in emotion recognition systems; Table 11 presents a summary of studies that evaluated gender differences in EEG emotion systems. These studies demonstrate various aspects of gender-specific responses in emotional and cognitive processes. These findings are compared with the results obtained through the proposed methodology. Several investigations have been conducted on the gender differences in emotional responses; however, no consistent findings have concluded which gender is more emotional. Studies [80,89] reported only a slight gender variation in regulation. Nolen et al. [90] demonstrated that women have higher usage of reshaping the situation and there is no significant gender difference in situations of suppression. In contrast, Parvaz et al. [91] proposed that no gender difference exists in LPP and Alpha frontal activity during cognitive reappraisal.

## 6. Conclusions and New Research Directions

The proposed methodology takes the first step, aiming to observe EEG data linked to guilt using a bottom-up experimental approach. This study addresses the research question of whether guilt and neutral emotions are related to human brain signals and investigates gender-based differences in responses to guilt and neutral emotional stimuli. The findings reveal that the brain not only has a connection to these emotions but also behaves distinctly in response to guilt and neutral emotions, with females showing greater expressiveness in response to emotional stimuli than male participants. Additionally, this contribution includes the development of stimuli to ignite guilt, further enhancing the understanding of its neural correlates. The findings of the proposed methodology illustrate the significance of EEG brain signals for the detection of complex emotions.

The classification results reveal that the classifier provides better accuracy with an engineered data set by bypassing the feature extraction process from EEG data. With the traditional approach, few EEG features seem to be specifically linked with the emotion of guilt. These features can be utilized to distinguish between guilt and neutral EEG data. Additionally all five major frequency bands have a pivotal role in distinguishing between guilt and non-guilt EEG data.Not only are the five major frequency bands significant, but critical information may also be present outside these bands, particularly in the case of complex emotions, as evident from better accuracy via the Data Engineering approach.

Moreover, the accuracies of both genders were analyzed for each band separately. It was revealed that female participants displayed higher accuracies across all bands except the Delta band. However, the limited sample size constrains the generalization of the findings, although it highlights that females are more attentive and expressive to emotional stimuli.

In the future, we plan to analyze more data processing techniques to compare the performance of proposed methodology. Finally, special efforts will made to make the collected data set available for further research to support human–computer interaction technology. Further, the proposed methodology will be improved by incorporating more data sets for training and testing.

## Figures and Tables

**Table 1:** Guilt-Inducing Events Collected from Participants.

Event	Description
1	**Friend Suffered Because of Me:** Feeling problems in an exam, I asked my friend for help. She got caught, her marks were deducted, and she lost her scholarship.
2	**Didn’t Perform Well in a Group Assignment:** I was unable to perform well in a group assignment, which was submitted late. As a result, every group member suffered because of me.
3	**Missed my Grandfather after His Demise:** I always thought of visiting him but kept delaying it to finish my chores. Unfortunately, he passed away while I kept postponing.
4	**Lied to My Mother Regarding Test Scores:** I got bad marks in my test and lied to my mother about them. She believed me.
5	**Damaged Someone’s Property:** At the mall, I played a shooting game and accidentally broke the gun. I returned it to the shopkeeper without admitting the damage.
6	**Bad Career Choice:** I got admission to two universities but chose one because my best friend joined it. Now, I regret not choosing the other option.
7	**Misbehaved with a Poor Person:** A beggar knocked on my door while I was watching a movie. Without listening to him, I scolded him.
8	**Being Rude to Parents:** My mother asked for my help, but I spoke to her very rudely.
9	**Refused to Help a Friend in Need:** A friend urgently needed my bike, but I lied about it having fuel issues to avoid helping.
10	**Prioritized Fun over Family Responsibilities:** My mother managed chaos at home alone because she thought I was studying at a friend’s place, but I was actually watching a movie.

**Table 2:** Neutral-Emotion Inducing Events.

Event	Description
1	**Going for a walk:** I ate a lot today, feeling that my abdomen was full. I went for a walk [55].
2	**An interaction with a stranger** [56].
3	**Riding a bicycle** [60].
4	**Went for shopping:** It was too cold. I didn’t have enough socks. I left for shopping [56].
5	**Having dinner at home:** I came late at home and asked for dinner.
6	**How to avoid COVID-19:** Wash your hands regularly.
7	**Having fun with a sibling:** Please don’t tell mother I broke the glass. Ok, then say ‘I am the best sibling in the world’.
8	**Hiring Bykea to go home:** I was done with my work then hired Bykea to go home.
9	**Discussion with a friend about yesterday’s lecture:** I was absent, please share the previous lecture with me.
10	**Daily routine life:** Start the day with exercise, then listening to music and mopping the house, and at the end some yoga.

**Table 3:** Data Samples’ Description for Classification—Data Engineering.

Data Samples	Description
Total Guilt Sample (GS)	876 samples
GS Dimension	868 by 16
GAHC Dimension	R × 868 by 16
Total Control Sample (CS)	516 samples
CS Dimension	868 by 16
CAHC Dimension	R × 868 by 16

**Table 4:** Randomly Selected Data Samples for Classification: Initial trial.

Sample	Randomly Selected Row	Sample Dimension
GAHC	32, 743, 817, 593, 661, 648, 342, 570, 149, 613, 28, 240, 40, 84, 710, 599	13,888 by 16
CAHC	144, 282, 492, 495, 81, 496, 488, 247, 406, 72, 213, 462, 399, 482, 329	13,888 by 16

**Table 5:** Initial trial Performance Metrics with 5-fold Cross-Validation.

5-Fold	Accuracy	F1 Score	Precision	Recall
**Average**	0.81	0.79	0.89	0.76

**Table 6:** Performance Metrics: Second Approach—Feature Extraction.

Accuracy	F1 Score	Precision	Recall
**0.63**	0.72	0.65	0.83

**Table 7:** Acquired Accuracy via Second Approach—Feature Extraction.

Calculated Features	Gamma γ	Beta β	Alpha α	Theta θ	Delta δ	All
Hjorth Activity	63%	60%	59%	62%	59%	60%
Hjorth Complexity	62.9%	61%	63%	63%	62.9%	62%
Hjorth Mobility	64%	63%	62.9%	62.9%	62%	64%
Band Power	61%	57%	58%	58%	58%	59%
Entropy	62.9%	63%	63%	62.9%	62.9%	63.5%
All Features	63%	64%	63.4%	62%	62%	63%

**Table 8:** Achieved Accuracy Across Different Frequency Bands for Males and Females using Feature Extraction Approach.

Band	Men (Accuracy)	Women (Accuracy)
**Gamma**	0.55399–0.6763	0.6115–0.7338
**Beta**	0.5755–0.6286	0.6115–0.7338
**Alpha**	0.5612–0.6763	0.6043–0.6906
**Theta**	0.5899–0.6331	0.5683–0.7143
**Delta**	0.5612–0.6691	0.5783–0.6244

**Table 9:** Summary of Studies Analyzing Guilt using Different Modalities.

Studies	Emotions	Modalities	Findings
Bhushan et al. [47]	Guilt	Facial clues	Neck touching (a gesture not a confirmed facial clue)
Julle et al. [48]	Guilt, Shame, Remorse	Thermal signals	Difference of 0.5 °C or more in face regions when guilt was felt, compared to shame and remorse.
Proposed Emotion Recognition System (1st Data Processing Approach)	Guilt	EEG signals	Accuracy of 63%
Proposed Emotion Recognition System (2nd Data Processing Approach)	Guilt	EEG signals	Accuracy of 83%

**Table 10:** Overview of Emotion Classification Systems Utilizing the Self-Collected Data Set.

Study	Emotions Explored	Number of Participants	Classifier	Findings
Sohaib et al. [84]	Valence, Arousal, Dominance	15 05	SVM SVM	56 % 66 %
Wang et al. [85]	Joy, Relaxation, Sadness, Fear	05	SVM	66.51%
Liu et al. [86]	Protection, Satisfaction, Delight, Surprise, Unhappiness, Feeling Unconcerned, Feeling Petrified, Annoyance	14	SVM	53.7%
Chen et al. [87]	Anger, Contempt, Sadness Disgust, Fear, Joy, Amazement	11	SVM KNN	35% 36%
Suhaimi et al. [88]	Calm, Happiness, Fear, Boredom	30	SVM	85%
Proposed Emotion Recognition System (1st Data Processing Approach)	Guilt	EEG signals	SVM	63%
Proposed Emotion Recognition System (2nd Data Processing Approach)	Guilt	EEG signals	SVM	83%

**Table 11:** Findings of Studies Investigating Gender Differences in Emotional Responses within EEG Emotion Systems.

Author	Finding
Li et al. [80]	Males are more responsive to positive environments, and females are more sensitive to discomforting environments.
Zhang et al. [92]	Females respond more quickly than males in low-level tasks, but not in high-level tasks.
Tolegenova et al. [93]	Females showed higher Theta values in the reappraisal condition.
Im et al. [94]	None.
Proposed Methodology	Female participants displayed higher accuracies across all bands except the Delta band.

## Data Availability

The original contributions presented in this study are included in the article. Further inquiries can be directed to the corresponding authors.
